# Exploring the influence of anemia and inflammation indices on colorectal cancer: analysis of the national health and nutrition examination survey From 2011 to 2018

**DOI:** 10.3389/fonc.2024.1457886

**Published:** 2024-09-03

**Authors:** Chao Qu, Shuting Yang, Tianli Shen, Qiuting Peng, Xuejun Sun, Yuyao Lin

**Affiliations:** ^1^ Department of General Surgery, The First Affiliated Hospital of Xi’an Jiaotong University, Shaanxi, Xi’an, China; ^2^ Department of Hepatobiliary Surgery, The First Affiliated Hospital of Xi’an Jiaotong University, Shaanxi, Xi’an, China; ^3^ Department of Plastic, Aesthetic and Maxillofacial Surgery, The First Affiliated Hospital of Xi’an Jiaotong University, Shaanxi, Xi’an, China

**Keywords:** colorectal cancer, NHANES, anemia, inflammation indices, risk

## Abstract

**Purpose:**

Patients with colorectal cancer (CRC) frequently present with anemia and signs of infection. However, the relationships between these factors remain unclear. This study investigated the potential association between anemia, inflammatory indices, and CRC.

**Methods:**

We analyzed data from the 2011–2018 National Health and Nutrition Examination Survey to investigate links between anemia, inflammation, and CRC. Inflammatory indices, including the neutrophil-percentage-to-albumin ratio, neutrophil-to-lymphocyte ratio, and eosinophil-to-lymphocyte ratio, were analyzed. Following rigorous inclusion criteria, 14,114 participants were included. Statistical methods such as logistic regression and subgroup analyses were employed. Moreover, survival analysis was performed.

**Results:**

Among the 14,114 participants, 0.6% had CRC and 11.0% were diagnosed with anemia. Anemia and inflammatory indices were associated with CRC, suggesting an increased risk (OR range: 2.03-2.50, P<0.05). Patients with CRC had lower red blood cell counts, reduced hemoglobin levels, and higher inflammatory indices. This is accompanied by an increase in the inflammatory indices, which is also a risk factor for CRC (OR range: 1.12-7.00, P<0.05). Survival analyses indicated that anemia was associated with lower survival rates, impacting all-cause, cancer, and CRC mortality.

**Conclusion:**

Our results indicate that anemia and inflammatory indices are correlated with CRC. Patients with CRC tend to exhibit increased inflammatory indices and decreased red blood cell count and albumin levels, potentially impacting survival.

## Introduction

Colorectal cancer (CRC) accounts for approximately 9.2% of all malignant neoplasms globally (1), making it the fourth deadliest cancer and the second leading cause of cancer-related mortality (2), with a survival rate of 14% (3). Age is a pivotal risk factor, linked to alterations in the intestinal microbiome and genetic predispositions (4). Although a range of determinants, including lifestyle choices and pre-existing health conditions, impact CRC prognosis, the influence of comorbidities, such as anemia, on CRC incidence and patient survival rates remains inadequately explored (5). Since anemia and inflammation are common and frequently evaluated indicators in patients with CRC, they can be practical tools for guiding clinical decisions and management strategies.

Anemia, defined as reduced hemoglobin levels or a diminished red blood cell count, affects approximately one-third of the global population (6). It is associated with increased morbidity and mortality rates, decreased productivity, and compromised neurodevelopment (7). Anemia is diagnosed when hemoglobin levels fall below 13 g/dL in males and below 12 g/dL in females (8). It frequently co-occurs with other diseases, markedly affecting survival outcomes and the prevalence of comorbidities, including infections and heart failure (9). Evidence links anemia with digestive system diseases, such as inflammatory bowel disease (10, 11) and cancer progression. Despite these associations, research on the relationship between anemia and digestive system malignancies, including CRC, is limited. Given the substantial health burden of CRC, it is imperative to explore its association with anemia (12).

Anemia is common in cancer patients because of red blood cell loss, increased destruction, and reduced functional red blood cells (13). The presence of anemia signifies a poor prognosis (14, 15) and is exacerbated by CRC treatments through surgery or chemotherapy, significantly affecting long-term outcomes (16, 17). These findings highlight the need for further research on the interplay between CRC and anemia.

A study exploring the prognostic significance of the hemoglobin, albumin, lymphocyte, and platelet (HALP) scores in solid tumors links low hemoglobin levels to adverse outcomes (18). The hemoglobin/erythrocyte distribution width ratio is a cost-effective, reproducible, and accessible prognostic marker for cancer survival (19). Additionally, in metastatic hormone-sensitive prostate cancer, low serum hemoglobin levels predict higher tumor-specific mortality, disease progression, and biochemical recurrence (20). Thus, hemoglobin levels are crucial for diagnosing anemia and assessing cancer risk.

The HALP score incorporates both anemia and inflammation markers. This study evaluated the prognostic impact of inflammatory biomarkers, specifically the neutrophil-percentage-to-albumin ratio (NPAR), neutrophil-to-lymphocyte ratio (NLR), and eosinophil-to-lymphocyte ratio (ELR), on long-term mortality in patients with CRC (6). While NPAR is a recognized inflammation indicator, few studies compare the prognostic significance of NPAR, NLR, and ELR in CRC. This study aimed to clarify these relationships (21).

We analyzed data from the National Health and Nutrition Examination Survey (NHANES) database to assess the association between inflammatory markers, hemoglobin levels, and CRC. Subgroup analysis was performed based on varying hemoglobin levels, ultimately establishing an association between anemia and inflammation on CRC. We also investigated the correlation between these factors and their impact on cancer survival.

## Materials and methods

### Study cohort

We utilized data from four NHANES cycles (2011–2018) to gather baseline and disease status information. Accessible on the Centers for Disease Control and Prevention (CDC) website (https://www.cdc.gov/nchs/nhanes/), the NHANES database provides extensive demographic, laboratory, and questionnaire data. This annual survey assesses the health and nutritional status of approximately 9,000 individuals in the U.S., covering demographic, economic, dietary, and health topics, along with medical, dental, physiological measurements, and laboratory tests. NHANES findings are essential for understanding disease prevalence and risk factors, facilitating health promotion and disease prevention efforts. All participants provided informed consent, and the NHANES received approval from the National Center for Health Statistics (NCHS) Research Ethics Review Board.

### CRC status

We accessed cancer status data from the Medical Conditions Questionnaire (MCQ) in the “Questionnaire Data” of the NHANES database at https://wwwn.cdc.gov/Nchs/Nhanes/2011-2012/MCQ_G.htm. This section provides self-reported health condition data for both children and adults. Specifically, information on malignant tumors was obtained through two questions: MCQ220: “Have you ever been told by a doctor or other health professional that you had a tumor or a malignancy?” and MCQ230: “What type of cancer was it?” Data collection involved interviews or computer-assisted systems with rigorous quality control measures implemented by trained professionals to minimize errors and ensure data accuracy.

### Diagnosis of anemia, hypertension, and diabetes

Participant data regarding anemia, hypertension, and diabetes status were obtained from various sources in the NHANES database. Laboratory data on anemia, including hemoglobin levels, were extracted from the “Standard Biochemical Profile” and the “Complete Blood Count and 5-Part Classification - Whole Blood” tests. Body measurements were collected from the “Body Measures” section. Information on hypertension and diabetes status was gathered from the “Blood Pressure & Cholesterol” and “Diabetes” questionnaire sections. Hypertension was defined as diastolic blood pressure >90 mmHg and/or systolic blood pressure >140 mmHg. Diabetes diagnosis relied on responses to the question “DIQ010: Has a doctor ever told you that you have diabetes?” Anemia was diagnosed according to the 1972 WHO standards, with thresholds of 130 g/L for men and 120 g/L for adult women (8).

### Mortality data

In 2020, the NCHS released the Public Use Mortality File for NHANES, covering 1999–2018. These files provide comprehensive information on mortality status and underlying causes, identified using the International Classification of Diseases, Tenth Revision (ICD-10). Individuals lacking sufficient identifying data or deemed ineligible for public release were excluded from our study. Our analyses specifically focused on data related to all-cause and cancer-related deaths.

### NPAR, NLR, and ELR

Neutrophil, lymphocyte, eosinophil, and albumin levels were obtained from basic biochemistry and complete blood counts (CBC). NPAR is calculated as the percentage of neutrophils (%) divided by serum albumin (g/dL), serving as a composite biomarker for neutrophils and albumin. NLR and ELR were calculated by dividing the absolute number of neutrophils by lymphocytes and eosinophils by lymphocytes, respectively. Additionally, the neutrophil percentage, representing the proportion of neutrophils in the white blood cell count, was determined. These biomarkers were derived from the same blood sample collected from each patient.

### Covariates

This study utilized seven groups of covariate types, categorized as follows: sociodemographic characteristics, including age, race (“non-Hispanic white,” “non-Hispanic black,” “Mexican American,” “Other Hispanic,” or “other race”), sex (“male” or “female”), education (“high school or below,” “college,” or “college graduate or above”), and body mass index (BMI); and habits of daily life, including drinking (“yes,” “no,” “don’t know,” or “not recorded”) and smoking (“yes,” “no,” or “don’t know”), as well as conditions such as diabetes (“yes,” “no,” or “don’t know”), hypertension (“yes,” “no,” or “don’t know”), coronary heart disease (“yes,” “no,” or “don’t know”), and stroke (“yes,” “no,” or “don’t know”).

### Statistical analysis

Statistical Product and Service Solutions (SPSS, version 26) software was used for all analyses. Continuous variables are presented as means ± standard deviations, and categorical variables as counts (percentages). Pearson’s chi-squared test was used to assess differences in categorical variables for anemia, inflammation, and cancer. T-tests were used for continuous variables. Chi-squared tests were used to investigate the associations between CRC, anemia, and inflammation, with odds ratios (ORs) and 95% confidence intervals (95% CI) calculated to quantify anemia’s risk impact. A two-tailed t-test (unpaired) or Mann-Whitney U test were used to analyze continuous variables. The χ^2^ test or Fisher’s exact test were used to analyze categorical variables.

We used multivariate logistic regression in SPSS to compute ORs and 95% CI, assessing the association between anemia and cancer. Seven models were calibrated to adjust for various cofounders: Model 0: no covariates were adjusted, Model 1 (age, race, sex, and education), Model 2 (smoking, drinking), Model 3 (BMI), Model 4 (diabetes), Model 5 (hypertension), Model 6 (coronary heart disease), and Model 7 (stroke). These adjustments corrected for potential confounding effects of anemia and inflammation indices on CRC. Covariates were selected based on previous studies to ensure the model validity and correction value.

We used the Kaplan–Meier method for survival analysis of patients with anemia, covering all-cause, cancer-specific, and CRC-specific mortality. Survival curves were plotted to illustrate these findings. Receiver operating characteristic (ROC) analyses were conducted to assess anemia’s predictive ability for mortality, shown as the area under the curve (AUC). Statistical significance was defined as a two-sided *P* value of <0.05.

## Results

### Study cohort selection

**Figure 1:**
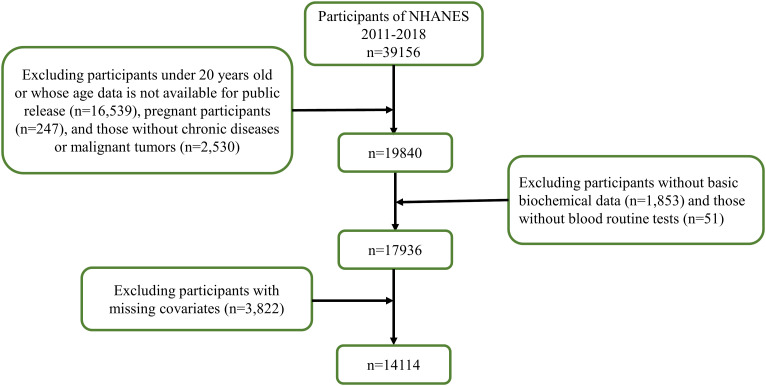
Flow chart of the screening process for the selection of eligible participants in NHANES 2011–2018.

In this study, we analyzed data from 2011 to 2018, comprising 39,156 participants. After excluding individuals under 20 years of age, pregnant women, and those with missing data on malignant tumors, hematological and biochemical tests, BMI, chronic diseases, and smoking and drinking habits, the final analysis included 14,114 individuals, as shown in **Figure 1**.

### Baseline characteristics of participants with CRC

**Table 1** shows that 91 CRC cases accounted for 0.6% of the total cohort (14,114). The average age at CRC diagnosis was 68.84 ± 12.47 years. CRC was associated with demographic factors such as age, race, marital status, smoking, and chronic diseases. Specifically, the proportion of CRC among non-Hispanic whites was 59.34%, significantly higher than that in other ethnic groups. Smoking and chronic diseases, such as high cholesterol, hypertension, and diabetes, were also linked to CRC. The data indicated that among CRC patients, 23.08% of them experience anemia, which is significantly higher than the probability of anemia in non-CRC individuals. Inflammatory indices, including NPAR, ELR, and NLR, were elevated. However, no differences in sex, alcohol consumption, or BMI were observed between the CRC and non-CRC groups (**Table 1**).

**Table 1:** Baseline characteristics of participants of with or not CRC.

Characteristic	Total n=14114 (100%)	With CRC n=91 (0.6%)	Without CRC n=14023 (99.4%)	P-value
Sex, n (%)				
Male	7445 (52.75)	45 (49.45)	7400 (53.77)	0.53
Female	6669 (47.25)	46 (50.55)	6623 (47.23)	
Age, Mean ± SE	49.52 ± 17.41	68.84 ± 12.47	49.39 ± 17.37	<0.001
Race, n (%)				
Mexican American	1825 (12.93)	6 (6.59)	1819 (12.97)	0.017
Other Hispanic	1383 (9.80)	8 (8.79)	1375 (9.81)	
Non-Hispanic White	5985 (42.40)	54 (59.34)	5931 (42.29)	
Non-Hispanic Black	2998 (21.24)	16 (17.58)	2982 (21.27)	
Other Race	1923 (13.62)	7 (7.69)	1916 (13.66)	
Education, n (%)				
Less than 9th grade	954 (6.76)	10 (10.99)	944 (6.73)	0.476
9-11th grade	1666 (11.80)	9 (9.89)	1657 (11.82)	
High school graduate	3195 (22.64)	23 (25.27)	3172 (22.62)	
College degree	4595 (32.56)	31 (34.07)	4564 (32.55)	
Above College	3699 (16.21)	18 (19.78)	3681 (26.25)	
Other	5 (0.04)	0 (0.00)	5 (0.03)	
Marital status, n (%)				
Married	7107 (50.35)	45 (49.45)	7062 (50.36)	<0.001
Widowed	969 (6.87)	23 (25.27)	946 (6.75)	
Divorced	1682 (11.92)	12 (13.19)	1670 (11.91)	
Separated	474 (3.36)	6 (6.59)	468 (3.34)	
Never married	2661 (18.85)	4 (4.40)	2657 (18.95)	
Living with partner	1219 (8.64)	1 (1.10)	1218 (8.69)	
Other	2 (0.01)	0 (0.00)	2 (0.01)	
Smoking, n (%)				
Yes	6857 (48.58)	59 (64.84)	6798 (48.48)	0.021
No	7247 (51.35)	32 (35.16)	7215 (51.45)	
Don’t know	10 (0.07)	0 (0.00)	10 (0.07)	
Drinking, n (%)				
Yes	2346 (16.62)	17 (18.68)	2329 (16.61)	0.596
No	11768 (83.38)	74 (81.32)	11694 (83.39)	
Hypertension, n (%)				
Yes	5255 (39.15)	54 (59.34)	5201 (37.09)	<0.001
No	8841 (62.64)	37 (40.66)	8804 (62.78)	
Don’t know	18 (0.13)	0 (0.00)	18 (0.13)	
CHD, n (%)				
Yes	607 (4.30)	12 (13.19)	595 (4.24)	<0.001
No	13464 (95.39)	79 (86.81)	13385 (95.45)	
Don’t know	43 (0.30)	0 (0.00)	43 (0.31)	
Stroke, n (%)				
Yes	552 (3.91)	9 (9.89)	543 (3.87)	0.012
No	13551 (96.01)	82 (90.11)	13469 (96.05)	
Don’t know	11 (0.08)	0 (0.00)	11 (0.08)	
Diabetes, n (%)				
Yes	1943 (13.77)	25 (27.47)	1918 (13.68)	0.002
No	11777 (83.44)	63 (69.23)	11714 (83.53)	
Don’t know	394 (2.79)	3 (3.30)	391 (2.79)	
BMI, Mean ± SE	29.50 ± 7.19	29.47 ± 6.02	29.50 ± 7.20	0.98
Anemia, n (%)				
Yes	1558 (11.04)	21 (23.08)	1537 (10.96)	<0.001
No	12556 (88.96)	70 (76.92)	12486 (89.04)	
NPAR, Mean ± SE	13.73 ± 2.61	14.68 ± 3.31	13.71 ± 2.60	<0.001
NLR, Mean ± SE	2.16 ± 1.23	2.47 ± 1.29	2.16 ± 1.22	0.023
ELR, Mean ± SE	0.10 ± 0.08	0.12 ± 0.08	0.10 ± 0.08	0.03

For continuous variables, numbers represent the mean ± standard deviation, and for categorical variables, numbers represent count (percentage).

### Baseline characteristics of participants with anemia

**Table 2** presents participant characteristics according to hemoglobin levels. Anemia definitions followed the WHO criteria: aged 15 years and older, no anemia (hemoglobin level ≥13 g/dL for males, ≥12 g/dL for females), mild anemia (11–12.9 g/dL for males, 11–11.9 g/dL for females), moderate anemia (8–10.9 g/dL for males and females), and severe anemia (<8 g/dL for males and females) (8). Our results showed that anemia affected 11.0% of the population, with a mean age of 55.44 ± 17.95 years. Anemia was more prevalent among females, older individuals, and non-Hispanic black individuals. It was also associated with chronic diseases and inflammatory indicators, but not with smoking or alcohol consumption.

**Table 2:** Baseline characteristics of participants of with or not anemia.

Characteristic	Total n=14114 (100%)	With anemia n=1558 (11.0%)	Without anemia n=12556 (89.0%)	P-value
Sex, n (%)				
Male	7445 (52.75)	626 (40.18)	6819 (64.31)	<0.001
Female	6669 (47.25)	932 (59.82)	5737 (45.69)	
Age, Mean ± SE	49.52 ± 17.41	55.44 ± 17.95	48.79 ± 17.20	<0.001
Race, n (%)				
Mexican American	1825 (12.93)	171 (10.98)	1654 (13.17)	<0.001
Other Hispanic	1383 (9.80)	126 (8.09)	1257 (10.01)	
Non-Hispanic White	5985 (42.40)	431 (27.60)	5554 (44.23)	
Non-Hispanic Black	2998 (21.24)	688 (44.16)	2310 (18.40)	
Other Race	1923 (13.62)	142 (9.11)	1781 (14.18)	
Education, n (%)				
Less than 9th grade	954 (6.76)	143 (9.18)	811 (6.46)	<0.001
9-11th grade	1666 (11.80)	218 (13.99)	1448 (11.53)	
High school graduate	3195 (22.64)	375 (24.07)	2820 (22.46)	
College degree	4595 (32.56)	497 (31.90)	4098 (32.64)	
Above College	3699 (16.21)	324 (20.80)	3375 (26.88)	
Other	5 (0.04)	1 (0.06)	4 (0.03)	
Marital status, n (%)				
Married	7107 (50.35)	714 (45.83)	6393 (50.92)	<0.001
Widowed	969 (6.87)	192 (12.32)	777 (6.19)	
Divorced	1682 (11.92)	206 (13.22)	1476 (11.76)	
Separated	474 (3.36)	68 (4.36)	406 (3.23)	
Never married	2661 (18.85)	286 (18.36)	2375 (18.92)	
Living with partner	1219 (8.64)	91 (5.84)	1128 (8.98)	
Other	2 (0.01)	1 (0.06)	1 (0.01)	
Smoking, n (%)				
Yes	6857 (48.58)	713 (45.76)	6144 (48.93)	0.119
No	7247 (51.35)	844 (54.17)	6403 (51.00)	
Don’t know	10 (0.07)	1 (0.06)	9 (0.07)	
Drinking, n (%)				
Yes	2346 (16.62)	268 (17.20)	2078 (16.55)	0.515
No	11768 (83.38)	1290 (82.80)	10478 (83.45)	
Hypertension, n (%)				
Yes	5255 (39.15)	820 (52.63)	4435 (35.32)	<0.001
No	8841 (62.64)	737 (47.30)	8104 (64.54)	
Don’t know	18 (0.13)	1 (0.06)	17 (0.14)	
CHD, n (%)				
Yes	607 (4.30)	123 (7.89)	484 (3.86)	<0.001
No	13464 (95.39)	1429 (91.72)	12035 (95.85)	
Don’t know	43 (0.30)	6 (0.39)	37 (0.29)	
Stroke, n (%)				
Yes	552 (3.91)	124 (7.96)	428 (3.41)	<0.001
No	13551 (96.01)	1433 (91.98)	12118 (96.51)	
Don’t know	11 (0.08)	1 (0.06)	10 (0.08)	
Diabetes, n (%)				
Yes	1943 (13.77)	373 (23.94)	1570 (12.50)	<0.001
No	11777 (83.44)	1137 (72.98)	10640 (84.74)	
Don’t know	394 (2.79)	48 (3.08)	346 (2.76)	
BMI, Mean ± SE	29.50 ± 7.19	30.24 ± 8.08	29.40 ± 7.07	<0.001
NPAR, Mean ± SE	13.73 ± 2.61	14.53 ± 3.24	13.63 ± 2.50	<0.001
NLR, Mean ± SE	2.16 ± 1.23	2.29 ± 1.57	2.15 ± 1.18	0.001
ELR, Mean ± SE	0.10 ± 0.08	0.11 ± 0.11	0.10 ± 0.08	<0.001

For continuous variables, numbers represent the mean ± standard deviation, and for categorical variables, numbers represent count (percentage).

### Correlation analysis between CRC and complete blood count

**Table 3** shows that CBC is crucial for diagnosing anemia and assessing inflammation. We classified the CBC results by CRC status and observed differences in red blood cell count (4.46 ± 0.52 & 4.69 ± 0.50 million cells/L, P<0.001) and hemoglobin level (13.45 ± 1.44 & 14.08 ± 1.52 g/dL, P<0.001) between patients with and without CRC, with corresponding differences in percentages. However, individual white blood cell count or percentages, notably neutrophils, did not show significant associations with CRC. The CBC changes observed in **Table 3** are closely related to anemia and inflammation.

**Table 3:** Correlation analysis between CRC and Complete Blood Count.

Complete Blood Count Mean ± SE	With CRC	Without CRC	P-value
Red blood cell count (million cells/L)	4.46 ± 0.52	4.69 ± 0.50	<0.001
Hemoglobin (g/dL)	13.45 ± 1.44	14.08 ± 1.52	<0.001
Hematocrit (%)	40.09 ± 4.06	41.73 ± 4.16	<0.001
Red cell distribution width (%)	13.97 ± 1.45	13.60 ± 1.32	0.007
Platelet count (1000 cells/μL)	227.12 ± 79.70	237.84 ± 61.79	0.1
Lymphocyte percent (%)	28.35 ± 9.42	30.71 ± 8.66	0.009
Monocyte percent (%)	8.89 ± 2.43	8.08 ± 2.33	0.001

For continuous variables, numbers represent the mean ± standard deviation.

### Association between CRC and anemia

**Figure 2:**
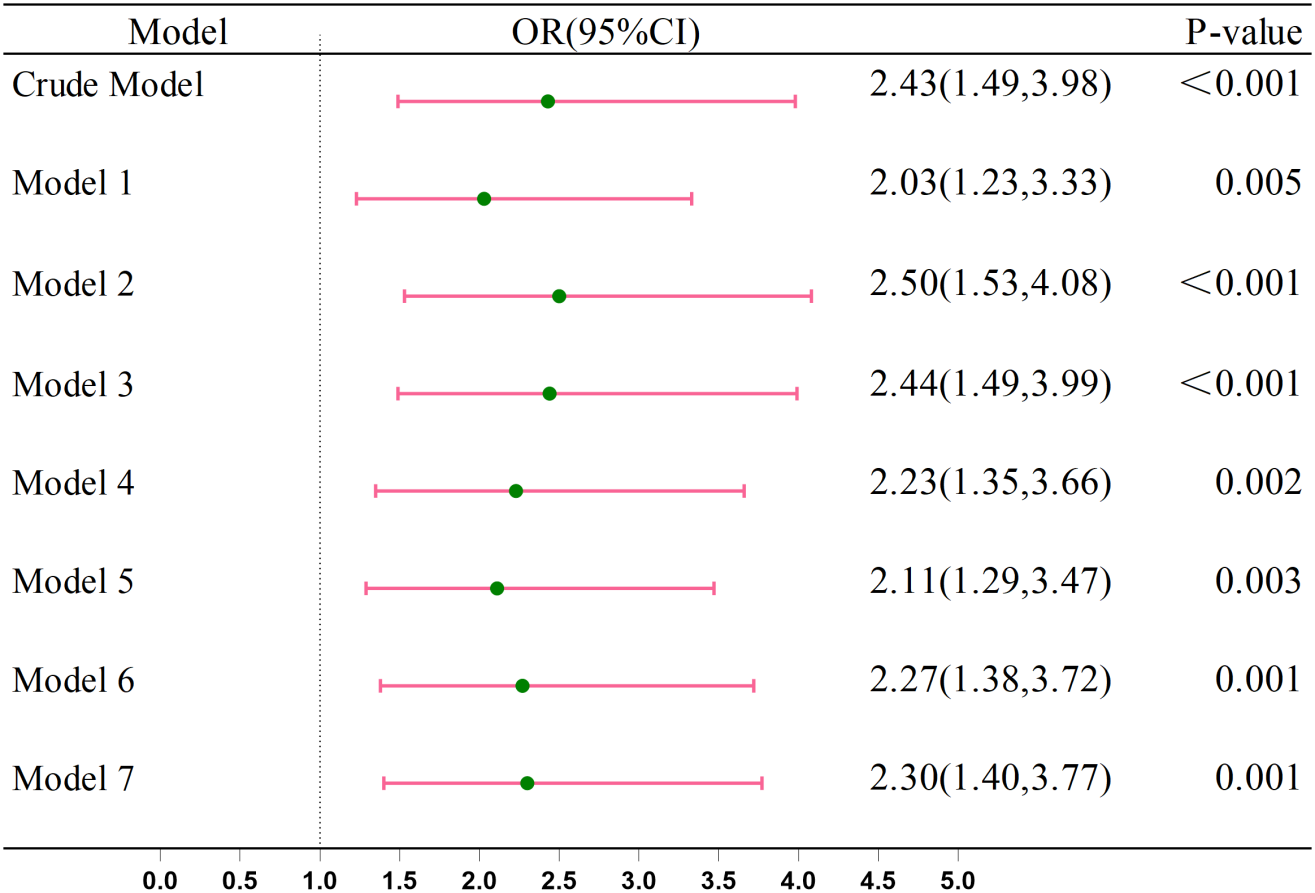
Association between the Anemia and CRC. Crude Model, without any adjustment. Model 1: Adjusted for age, race, gender, and education. Model 2: Further adjusted for smoking and drinking habits. Model 3: Included adjustment for BMI. Model 4: Included adjustment for diabetes. Model 5: Included adjustment for hypertension. Model 6: Included adjustment for coronary heart disease. Model 7: Included adjustment for stroke.

**Figure 2** shows the integration of seven covariate models into the crude model to assess anemia’s relationship with CRC. Before adjustment, the OR was 2.43 (95% CI: 1.49–3.98). After adjustment, the OR values were as follows: sex, age, race, and education [OR = 2.03 (95% CI: 1.23–3.33)], smoking and drinking [OR = 2.50 (95% CI: 1.53–4.08)], BMI [OR = 2.44 (95% CI: 1.49–3.99)], diabetes [OR = 2.23 (95% CI: 1.35–3.66)], hypertension [OR = 2.11 (95% CI: 1.29–3.47)], coronary heart disease [OR = 2.27 (95% CI: 1.38–3.72)], and stroke [OR = 2.30 (95% CI: 1.40–3.77)]. All adjusted ORs were >1, indicating an increased CRC risk with anemia in multivariate logistic regression analysis.

### Association between NPAR, NLR, and ELR and CRC, and their effects on the association of anemia with CRC

**Table 4** shows the relationships between NPAR, NLR, ELR, and CRC. Patients with CRC generally exhibited higher inflammatory indicator values. Subsequently, after categorizing into four quartiles, NPAR was classified as follows: Q1 (<12.02), Q2 (12.02–13.66), Q3 (13.67–15.40), and Q4 (≥15.40); for NLR, Q1 (1.44), Q2 (1.44–1.93), Q3 (1.93–2.66), and Q4 (≥2.66); and for ELR, Q1 (<0.05), Q2 (0.05–0.07), Q3 (0.07–0.13), and Q4 (≥0.13). Across all quartiles, the proportion of patients with CRC was higher than those without CRC. Subgroup analysis indicated a higher proportion of patients with CRC in the Q3 and Q4 quartiles compared to Q1 and Q2, particularly with ELR exceeding 70% in Q3 and Q4.

**Table 4:** Association between CRC and NPAR, NLP and ELR.

Characteristics	With CRC	Without CRC	P-value
**NPAR**	14.68 ± 3.31	13.71 ± 2.60	<0.001
Q1 (<12.02)	17 (18.68)	3499 (24.95)	0.073
Q2 (12.02-13.66)	18 (19.78)	3535 (25.21)	
Q3 (13.66-15.4)	24 (26.37)	3591 (25.61)	
Q4 (>15.4)	32 (35.16)	3398 (24.23)	
**NLR**	2.47 ± 1.29	2.16 ± 1.22	0.001
Q1 (<1.44)	17 (18.68)	3487 (24.87)	0.046
Q2 (1.44-1.93)	22 (24.18)	3554 (25.34)	
Q3 (1.93-2.57)	18 (19.78)	3489 (24.88)	
Q4 (>2.57)	34 (37.36)	3493 (24.91)	
**ELR**	0.12 ± 0.08	0.10 ± 0.08	<0.001
Q1 (<0.05)	16 (17.58)	3338 (23.80)	0.027
Q2 (0.05-0.07)	11 (12.08)	2797 (19.95)	
Q3 (0.07-0.13)	32 (35.16)	4516 (32.20)	
Q4 (>0.13)	32 (35.16)	3372 (24.05)	

For continuous variables, numbers represent the mean ± standard deviation, and for categorical variables, numbers represent count (percentage).

**Figure 3:**
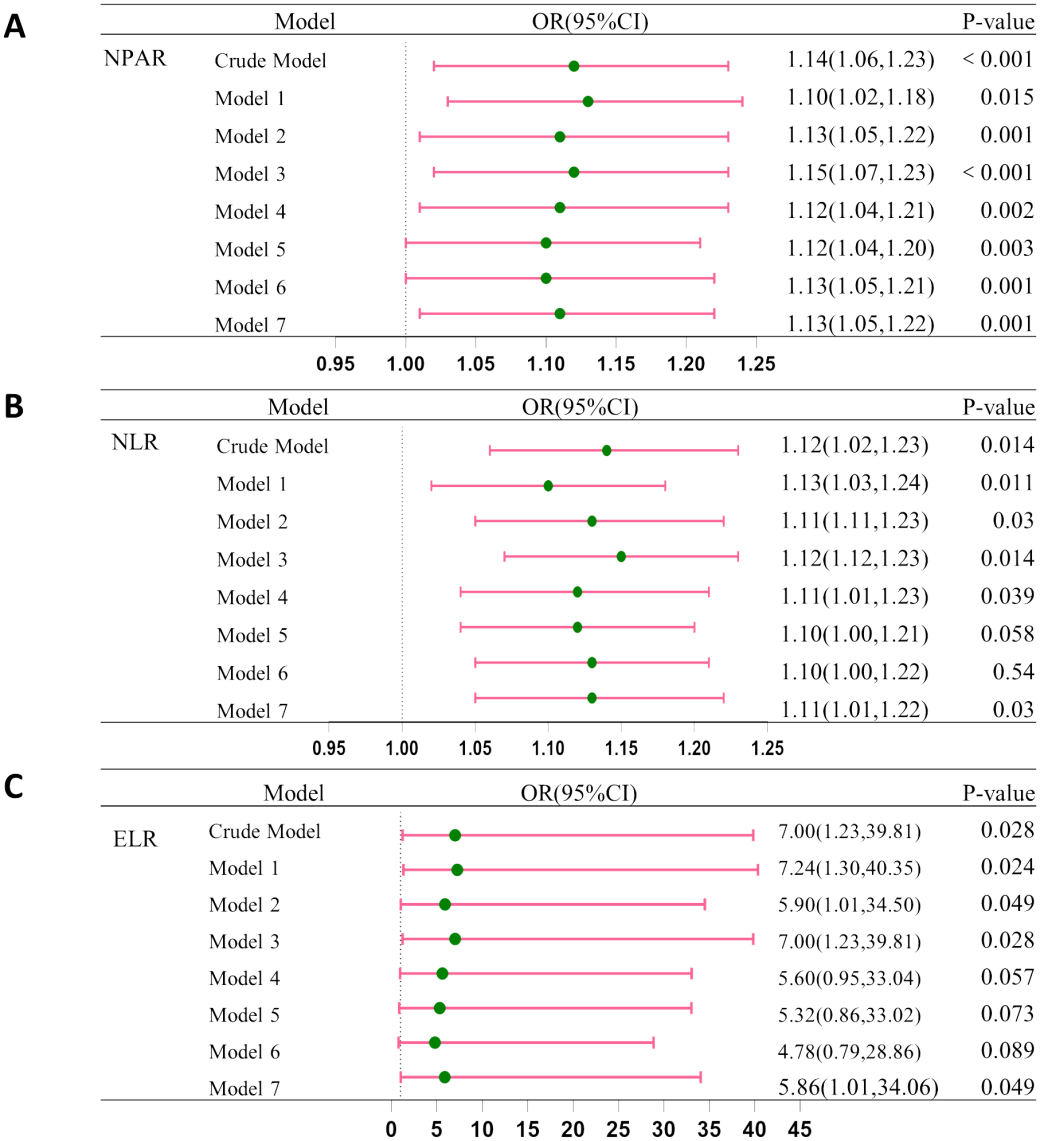
Association between CRC and **(A)** NPAP, **(B)** NLR and **(C)** ELR. Crude Model, without any adjustment; Model 1: Adjusted for age, race, gender, and education. Model 2: Further adjusted for smoking and drinking habits. Model 3: Included adjustment for BMI. Model 4: Included adjustment for diabetes. Model 5: Included adjustment for hypertension. Model 6: Included adjustment for coronary heart disease. Model 7: Included adjustment for stroke.

**Figure 3** shows the integration of these models to examine the relationship between inflammatory indicators (NPAR, NLR, and ELR), and CRC. Before adjustment, ORs were: NPAR [OR = 1.14 (95% CI: 1.06–1.23)], NLR [OR = 1.12 (95% CI: 1.02–1.23)], and ELR [OR = 7.00 (95% CI: 1.23–39.81)]. After adjustment most indicators suggested elevated CRC risk, suggesting the potential role of inflammatory markers in CRC development.

**Figure 4:**
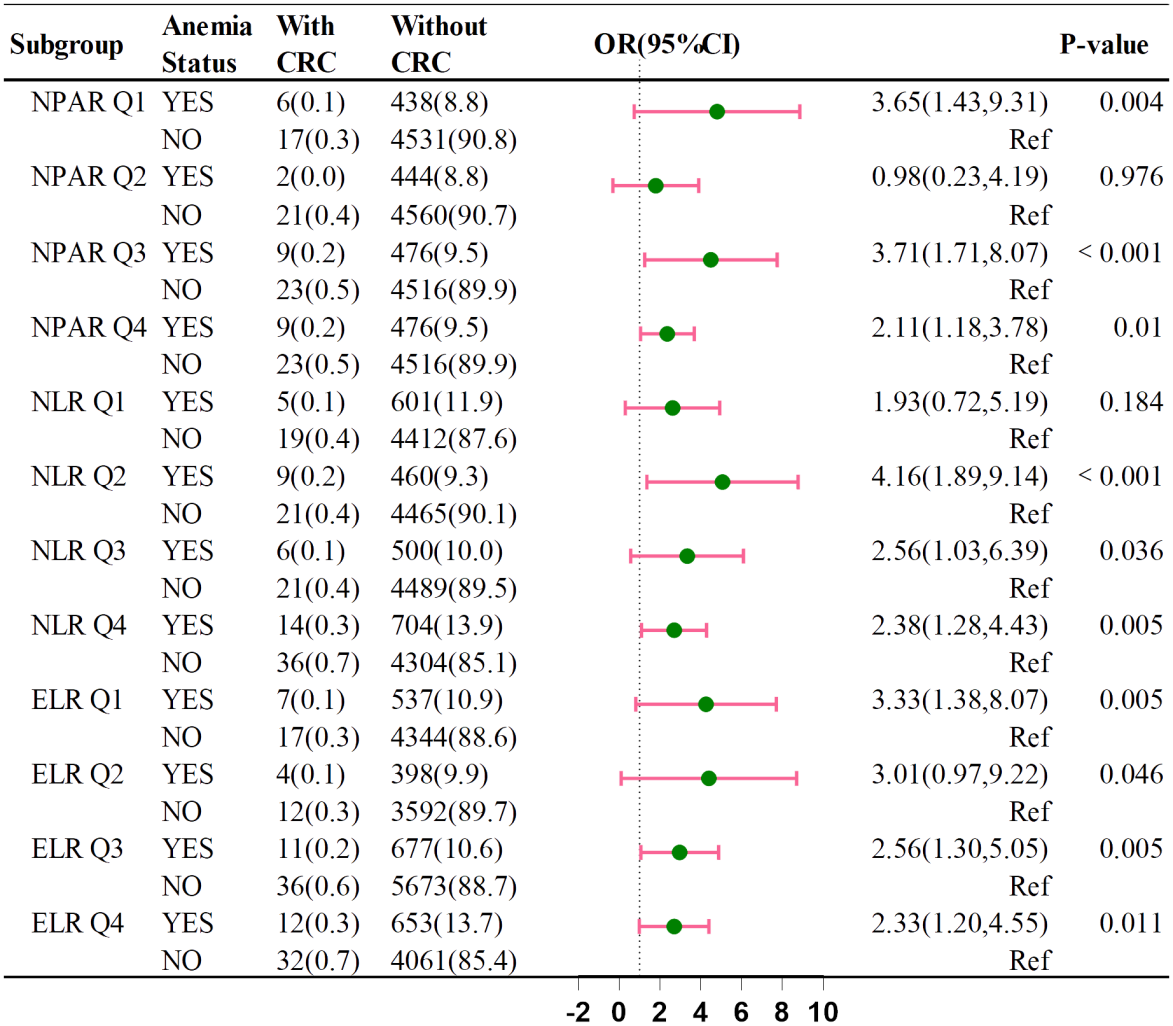
Association between the anemia on CRC by NPAP, NLR and ELR.

**Figure 4** shows that anemia’s impact on CRC risk varies across NPAR, NLR, and ELR subgroups. The ORs were: NPAR Q1 (OR = 3.07, 95% CI: 1.01–9.37), NPAR Q3 (OR = 3.82, 95% CI: 1.59–9.14), NLR Q2 (OR = 3.01, 95% CI: 1.12–8.11), NLR Q4 (OR = 2.39, 95% CI: 1.11–5.16), ELR Q2 (OR = 3.55, 95% CI: 0.95–13.32), and ELR Q3 (OR = 2.50, 95% CI: 1.09–5.75). These indicators suggest that anemia may increase CRC risk within the four categories of NPAR, NLR, and ELR.

### Anemia and cancer mortality outcomes

**Figure 5:**
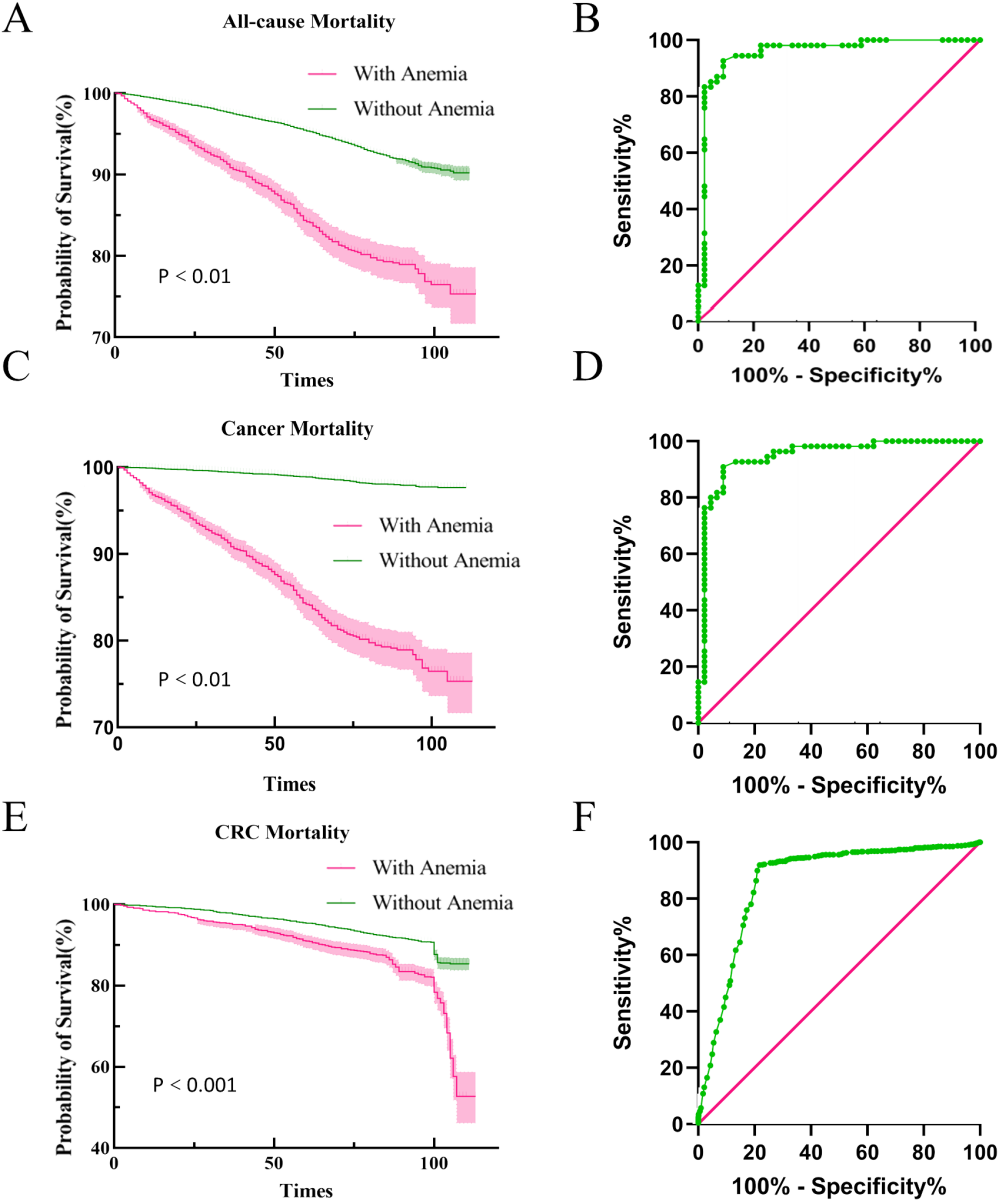
Kaplan-Meier survival curves for mortality outcomes. All-cause and cancer mortality and ROC curves **(A, B)** All-cause mortality survival curve and ROC curve for all-cause mortality, **(C, D)** Cancer mortality survival curve and ROC curve for cancer mortality, **(E, F)** CRC mortality survival curve and ROC curve for CRC mortality.

In our analysis of follow-up outcomes and death data from 2011 to 2018, we aimed to construct survival curves and authenticate all-cause and cancer-specific mortality data in patients with CRC. After adjusting for potential confounders, individuals with anemia had significantly poorer survival outcomes than those without anemia across all measured endpoints. The area under the ROC curve indicated a significant difference in cancer-related mortality between those with and without anemia (**Figure 5**). These findings underscore the negative influence of anemia on cancer prognosis and mortality.

## Discussion

This cohort study, spanning from 2011 to 2018, utilized comprehensive questionnaire information reflecting the demographics of the general U.S. population. Our results show an association between anemia, inflammatory markers (NPAR, ELR, and NLR), and CRC. Statistical analysis indicated that anemia and higher levels of these markers increased the risk of CRC. Hematological analysis revealed notable disparities in red blood cell-related indicators, with a higher prevalence of anemia in patients with CRC, especially older individuals (22). Cancer and chemotherapy-induced anemia are common (23), with prevalence rates between 30% and 90% (23, 24). Subgroup analysis of hemoglobin levels highlighted distinct distributions in patients with CRC. Moreover, survival curve analysis revealed increased cancer mortality rates among patients with anemia, spanning multiple causes of death. Despite the clinical significance of this association, research on the relationship between CRC and anemia remains limited, impacting patient quality of life and prognosis (25). Further investigation is needed to understand this relationship and inform clinical management strategies.

Anemia is more prevalent in advanced stages of CRC (26), with survival curve analysis showing elevated cancer mortality rates among patients with anemia across various causes of death. Anemia not only diminishes the quality of life of patients with cancer but also negatively impacts cancer prognosis, potentially increasing fivefold (27).

Anemia in patients with CRC is often due to gastrointestinal bleeding or iron deficiency (28). Our study observed an increased risk of CRC associated with anemia, leading to delayed treatment and worse survival (29). After adjusting for covariates, combining analysis of anemia with NPAR, ELR, and NLR showed a predictive effect on CRC risk. Simple blood tests can determine the presence of anemia or elevated NPAR, ELR, or NLR. Further prospective studies are necessary to establish a direct relationship between these indicators and CRC. This novel finding sheds light on an underexplored aspect of the association between CRC and anemia, suggesting that this association may disrupt the intestinal microbiota and influence cancer progression (30). Further research is warranted to elucidate these relationships and their implications for clinical practice and public health interventions.

Inflammation plays a pivotal role in both CRC and CRC with anemia. Our evaluation of CRC using inflammatory markers (NPAR, NLR, and ELR) revealed that elevated markers correlated with increased risk. NPAR, a measurable inflammatory indicator (31), has a significant predictive value in the prognosis of various malignant tumors (32, 33). Similarly, NLR has been linked to systemic inflammation in critically ill patients (34) and elevated cancer risk and mortality (35, 36). Studies have demonstrated an association between NLR and cancer risk (37), along with its impact on overall survival in patients with non-small cell lung cancer (38). Elevated ELR may indicate a poor cancer prognosis (39, 40). Integrating these biochemical indicators enhances prognostic accuracy for patients with anemic CRC, aiding in better understanding and management of CRC with anemia.

In addition to anemia and inflammation indices, there are now other methods used for the diagnosis of CRC, and deep learning (DL) is one of them. DL is a subtype of artificial intelligence (AI), and convolutional neural networks (CNN) is a form of supervised DL with multiple layers of artificial neurons. Scientists have trained a CNN to identify malignant tumors and achieve accurate diagnoses (41). In this way, DL can help researchers identify CRC more efficiently and accurately (42).

It is crucial to acknowledge the limitations of our study, particularly regarding the analysis of hemoglobin levels, a critical indicator of anemia. We did not specifically investigate severe anemia (hemoglobin < 6.0 g/dL), which may be more prevalent among patients with cancer and significantly impact those with CRC. Additionally, our study’s observational nature means residual confounding factors cannot be entirely ruled out (43).

Future research should include a more comprehensive analysis of hemoglobin levels, particularly severe anemia, and further explore the mechanistic links between anemia and CRC development. Understanding how cancer, chronic infections, and inflammation contribute to anemia could lead to therapeutic interventions targeting anemia in patients with CRC, ultimately improving outcomes. Preventing and correcting anemia could potentially improve survival for individuals with CRC. Further studies and clinical trials are needed to explore strategies for managing anemia in patients with CRC to improve survival and quality of life.

## Conclusion

Our study highlights the association between anemia, inflammatory indices, and CRC, suggesting that anemia and inflammation may be significant risk indicators in patients with CRC. Patients with anemia have lower survival rates, underscoring the urgent need to address anemia in CRC management. Early detection and effective management of anemia are essential for improving the prognosis and quality of life of patients with CRC. These findings emphasize the importance of continued research and clinical efforts to understand and address the complexity of anemia in CRC, ultimately improving patient care and outcomes.

## Data Availability

The original contributions presented in the study are included in the article/supplementary material. Further inquiries can be directed to the corresponding author/s.

## References

[1] Siegel RL Wagle NS Cercek A Smith RA Jemal A. Colorectal cancer statistics, 2023. CA Cancer J Clin. (2023) 73:233–54. doi: 10.3322/caac.21772 36856579

[2] Dekker E Tanis PJ Vleugels J Kasi PM Wallace MB. Colorectal cancer. Lancet. (2019) 394:1467–80. doi: 10.1016/S0140-6736(19)32319-0 31631858

[3] Li J Ma X Chakravarti D Shalapour S DePinho RA. Genetic and biological hallmarks of colorectal cancer. Genes Dev. (2021) 35:787–820. doi: 10.1101/gad.348226.120 34074695 PMC8168558

[4] Yimin E Lu C Zhu K Li W Sun J Ji P. Function and mechanism of exosomes derived from different cells as communication mediators in colorectal cancer metastasis. iScience. (2024) 27:109350. doi: 10.1016/j.isci.2024.109350 38500820 PMC10945197

[5] Shin AE Giancotti FG Rustgi AK. Metastatic colorectal cancer: mechanisms and emerging therapeutics. Trends Pharmacol Sci. (2023) 44:222–36. doi: 10.1016/j.tips.2023.01.003 PMC10365888 36828759

[6] Yao J Chen X Meng F Cao H Shu X. Combined influence of nutritional and inflammatory status and depressive symptoms on mortality among US cancer survivors: Findings from the NHANES. Brain Behav Immun. (2024) 115:109–17. doi: 10.1016/j.bbi.2023.10.002 37820973

[7] Chaparro CM Suchdev PS. Anemia epidemiology, pathophysiology, and etiology in low- and middle-income countries. Ann N Y Acad Sci. (2019) 1450:15–31. doi: 10.1111/nyas.14092 31008520 PMC6697587

[8] Stauder R Valent P Theurl I. Anemia at older age: etiologies, clinical implications, and management. Blood. (2018) 131:505–14. doi: 10.1182/blood-2017-07-746446 29141943

[9] Gallagher PG. Anemia in the pediatric patient. Blood. (2022) 140:571–93. doi: 10.1182/blood.2020006479 PMC9373018 35213686

[10] Gomollón F Gisbert JP. Anemia and digestive diseases: an update for the clinician. World J Gastroenterol. (2009) 15:4615–6. doi: 10.3748/wjg.15.4615 PMC2754508 19787823

[11] Yang S Li X Jiang Z. The interaction of perfluoroalkyl acids and a family history of diabetes on arthritis: analyses of 2011-2018 NHANES. BMC Public Health. (2024) 24:448. doi: 10.1186/s12889-024-17879-2 38347551 PMC10863084

[12] Cascio MJ DeLoughery TG. Anemia: evaluation and diagnostic tests. Med Clin North Am. (2017) 101:263–84. doi: 10.1016/j.mcna.2016.09.003 28189170

[13] Dicato M Plawny L Diederich M. Anemia in cancer. Ann Oncol. (2010) 21 Suppl 7:vii167–172. doi: 10.1093/annonc/mdq284 20943610

[14] Yuan T Jia Q Zhu B Chen D Long H. Synergistic immunotherapy targeting cancer-associated anemia: prospects of a combination strategy. Cell Commun Signal. (2023) 21:117. doi: 10.1186/s12964-023-01145-w 37208766 PMC10199626

[15] Gilreath JA Rodgers GM. How I treat cancer-associated anemia. Blood. (2020) 136:801–13. doi: 10.1182/blood.2019004017 32556170

[16] Moncur A Chowdhary M Chu Y Francis NK. Impact and outcomes of postoperative anaemia in colorectal cancer patients: a systematic review. Colorectal Dis. (2021) 23:776–86. doi: 10.1111/codi.15461 33249731

[17] Keeler BD Dickson EA Simpson JA Ng O Padmanabhan H Brookes MJ. The impact of pre-operative intravenous iron on quality of life after colorectal cancer surgery: outcomes from the intravenous iron in colorectal cancer-associated anaemia (IVICA) trial. Anaesthesia. (2019) 74:714–25. doi: 10.1111/anae.14659 30963552

[18] Xu H Zheng X Ai J Yang L. Hemoglobin, albumin, lymphocyte, and platelet (HALP) score and cancer prognosis: A systematic review and meta-analysis of 13,110 patients. Int Immunopharmacol. (2023) 114:109496. doi: 10.1016/j.intimp.2022.109496 36462339

[19] Coradduzza D Medici S Chessa C Zinellu A Madonia M Angius A. Assessing the predictive power of the hemoglobin/red cell distribution width ratio in cancer: A systematic review and future directions. Medicina (Kaunas). (2023) 59:2124. doi: 10.3390/medicina59122124 38138227 PMC10744746

[20] Mori K Janisch F Mostafaei H Lysenko I Karakiewicz PI Enikeev DV. Prognostic value of hemoglobin in metastatic hormone-sensitive prostate cancer: A systematic review and meta-analysis. Clin Genitourin Cancer. (2020) 18:e402–9. doi: 10.1016/j.clgc.2019.12.002 32007439

[21] Lan C-C Su W-L Yang M-C Chen S-Y Wu Y-K. Predictive role of neutrophil-percentage-to-albumin, neutrophil-to-lymphocyte and eosinophil-to-lymphocyte ratios for mortality in patients with COPD: Evidence from NHANES 2011-2018. Respirology. (2023) 28:1136–46. doi: 10.1111/resp.14589 37655985

[22] Gilreath JA Stenehjem DD Rodgers GM. Diagnosis and treatment of cancer-related anemia. Am J Hematol. (2014) 89:203–12. doi: 10.1002/ajh.23628 24532336

[23] Abdel-Razeq H Hashem H. Recent update in the pathogenesis and treatment of chemotherapy and cancer induced anemia. Crit Rev Oncol Hematol. (2020) 145:102837. doi: 10.1016/j.critrevonc.2019.102837 31830663

[24] Rodgers GM 3rd Becker PS Blinder M Cella D Chanan-Khan A Cleeland C. Cancer- and chemotherapy-induced anemia. J Natl Compr Canc Netw. (2012) 10:628–53. doi: 10.6004/jnccn.2012.0064 22570293

[25] Laï-Tiong F Brami C Dubroeucq O Scotté F Curé H Jovenin N. Management of anemia and iron deficiency in a cancer center in France. Support Care Cancer. (2016) 24:1091–6. doi: 10.1007/s00520-015-2877-4 26253586

[26] Väyrynen JP Tuomisto A Väyrynen SA Klintrup K Karhu T Mäkelä J. Preoperative anemia in colorectal cancer: relationships with tumor characteristics, systemic inflammation, and survival. Sci Rep. (2018) 8:1126. doi: 10.1038/s41598-018-19572-y 29348549 PMC5773501

[27] Kenar G Köksoy EB Ürün Y Utkan G. Prevalence, etiology and risk factors of anemia in patients with newly diagnosed cancer. Support Care Cancer. (2020) 28:5235–42. doi: 10.1007/s00520-020-05336-w 32086566

[28] Krieg S Loosen S Krieg A Luedde T Roderburg C Kostev K. Association between iron deficiency anemia and subsequent stomach and colorectal cancer diagnosis in Germany. J Cancer Res Clin Oncol. (2024) 150:53. doi: 10.1007/s00432-023-05534-z 38289465 PMC10827837

[29] Sharma P Georgy JT Andrews AG John AO Joel A Chacko RT. Anemia requiring transfusion in breast cancer patients on dose-dense chemotherapy: Prevalence, risk factors, cost and effect on disease outcome. Support Care Cancer. (2022) 30:5519–26. doi: 10.1007/s00520-022-06970-2 PMC8937495 35314996

[30] Shi X-Q Zhu Z-H Yue S-J Tang Y-P Chen Y-Y Pu Z-J. Studies on blood enrichment and anti-tumor effects of combined Danggui Buxue Decoction, Fe and rhEPO based on colon cancer-related anemia model and gut microbiota modulation. Chin J Nat Med. (2021) 19:422–31. doi: 10.1016/S1875-5364(21)60041-9 34092293

[31] Liu CF Chien LW. Predictive role of neutrophil-percentage-to-albumin ratio (NPAR) in nonalcoholic fatty liver disease and advanced liver fibrosis in nondiabetic US adults: evidence from NHANES 2017-2018. Nutrients. (2023) 15:1892–907. doi: 10.3390/nu15081892 PMC10141547 37111111

[32] Ko C-A Fang K-H Tsai M-S Lee Y-C Lai C-H Hsu C-M. Prognostic value of neutrophil percentage-to-albumin ratio in patients with oral cavity cancer. Cancers (Basel). (2022) 14:4892. doi: 10.3390/cancers14194892 36230814 PMC9564168

[33] Tang Y Hou H Li L Yong L Zhang S Yan L. Neutrophil percentage-to-albumin ratio: A good parameter for the evaluation of the severity of anti-NMDAR encephalitis at admission and prediction of short-term prognosis. Front Immunol. (2022) 13:847200. doi: 10.3389/fimmu.2022.847200 35479085 PMC9035690

[34] Cupp MA Cariolou M Tzoulak I Aune D Evangelou E Berlanga-Taylor AJ. Neutrophil to lymphocyte ratio and cancer prognosis: an umbrella review of systematic reviews and meta-analyses of observational studies. BMC Med. (2020) 18:360. doi: 10.1186/s12916-020-01817-1 33213430 PMC7678319

[35] Nøst TH Alcala K Urbarova U Byrne KS Guida F Sandanger TM. Systemic inflammation markers and cancer incidence in the UK Biobank. Eur J Epidemiol. (2021) 36:841–8. doi: 10.1007/s10654-021-00752-6 PMC8416852 34036468

[36] Mukaida N Sasaki SI Baba T. Two-faced roles of tumor-associated neutrophils in cancer development and progression. Int J Mol Sci. (2020) 21:3457–79. doi: 10.3390/ijms21103457 PMC7278934 32422991

[37] Chung C Seo W Silwal P Jo EK. Crosstalks between inflammasome and autophagy in cancer. J Hematol Oncol. (2020) 13:100. doi: 10.1186/s13045-020-00936-9 32703253 PMC7376907

[38] Diem S Schmid S Krapf M Flatz L Born D Jochum W. Neutrophil-to-Lymphocyte ratio (NLR) and Platelet-to-Lymphocyte ratio (PLR) as prognostic markers in patients with non-small cell lung cancer (NSCLC) treated with nivolumab. Lung Cancer. (2017) 111:176–81. doi: 10.1016/j.lungcan.2017.07.024 28838390

[39] Holub K Biete A. Impact of systemic inflammation biomarkers on the survival outcomes of cervical cancer patients. Clin Transl Oncol. (2019) 21:836–44. doi: 10.1007/s12094-018-1991-4 30470994

[40] Holub K Biete A. New pre-treatment eosinophil-related ratios as prognostic biomarkers for survival outcomes in endometrial cancer. BMC Cancer. (2018) 18:1280. doi: 10.1186/s12885-018-5131-x 30577833 PMC6304088

[41] Bousis D Verras GI Bouchagier K Antzoulas A Panagiotopoulos I Katinioti A. The role of deep learning in diagnosing colorectal cancer. Prz Gastroenterol. (2023) 18:266–73. doi: 10.5114/pg.2023.129494 PMC10626379 37937113

[42] Chlorogiannis DD Verras GI Tzelepi V Chlorogiannis A Apostolos A Kotis K. Tissue classification and diagnosis of colorectal cancer histopathology images using deep learning algorithms. Is the time ripe for clinical practice implementation. Prz Gastroenterol. (2023) 18:353–67. doi: 10.5114/pg.2023.130337 PMC10985751 38572457

[43] Ying H Gao L Liao L Xu X Yu W Hong W. Association between niacin and mortality among patients with cancer in the NHANES retrospective cohort. BMC Cancer. (2022) 22:1173. doi: 10.1186/s12885-022-10265-4 36376861 PMC9661743

